# ERH Gene and Its Role in Cancer Cells

**DOI:** 10.3389/fonc.2022.900496

**Published:** 2022-05-23

**Authors:** Kun Pang, Mei-li Li, Lin Hao, Zhen-duo Shi, Harry Feng, Bo Chen, Yu-yang Ma, Hao Xu, Deng Pan, Zhe-Sheng Chen, Cong-hui Han

**Affiliations:** ^1^ Department of Urology, Xuzhou Central Hospital, Affiliated Central Hospital of Xuzhou Medical University, Xuzhou, China; ^2^ Department of Ophthalmology, The Affiliated Xuzhou Municipal Hospital of Xuzhou Medical University, Xuzhou First People's Hospital, Xuzhou, China; ^3^ Department of Ophthalmology, Eye Disease Prevention and Treatment Institute of Xuzhou, Xuzhou, China; ^4^ STEM Academic Department, Wyoming Seminary, Kingston, PA, United States; ^5^ Graduate School, Bengbu Medical College, Bengbu, China; ^6^ College of Pharmacy and Health Sciences, St. John’s University, New York, NY, United States

**Keywords:** enhancer of rudimentary homolog (ERH) gene, oncogenesis factor, protein partner, transcription factor, tumor-targeted therapy

## Abstract

Cancer is a major public health problem worldwide. Studies on oncogenes and tumor-targeted therapies have become an important part of cancer treatment development. In this review, we summarize and systematically introduce the gene enhancer of rudimentary homolog (ERH), which encodes a highly conserved small molecule protein. ERH mainly exists as a protein partner in human cells. It is involved in pyrimidine metabolism and protein complexes, acts as a transcriptional repressor, and participates in cell cycle regulation. Moreover, it is involved in DNA damage repair, mRNA splicing, the process of microRNA hairpins as well as erythroid differentiation. There are many related studies on the role of ERH in cancer cells; however, there are none on tumor-targeted therapeutic drugs or related therapies based on the expression of ERH. This study will provide possible directions for oncologists to further their research studies in this field.

Cancer is a major public health problem worldwide and is the second leading cause of death in the United States: 1,918,030 new cancer cases and 609,360 cancer deaths are projected to occur in the United States in 2022 (1). However, with the advancement of cancer treatment strategies, cancer mortality has continued to decline since 1991 (2). There have been more and more studies on oncogenes, and tumor-targeted therapies have become an important part of many cancer treatment options (3). Targeted therapies have changed the systemic treatment options for cancer patients. To further prolong the survival time and improve the quality of life of cancer patients, studies on oncogenes for tumor-targeted therapies are ongoing. Recently, more and more studies have shown that the expression of enhancer of rudimentary homolog (ERH) gene is closely related to cancers (4, 5). In this review, we summarize and systematically introduce the ERH gene and its role in cancer cells.

In 2007, Jin T *et al.* first started to suspect and discover the relationship between ERH and malignant tumors (6). They found that the ERH protein was downregulated in von Hippel-Lindau (VHL) tumors with Y98H mutation, but ERH expression was upregulated in many other metastatic tumors. They did not clarify whether the difference in ERH expression was the cause or the result of the VHL tumor. In 2008, Zakrakas M *et al.* found in their study (7) that comparing with non-tumorigenic breast cancer and normal breast tissue samples, ERH expression is upregulated in tumorigenic cell lines. They also found in ovarian cancer cell lines that, ERH is clearly upregulated with tumor progression. They considered that ERH could be used clinically as a prognostic factor in breast and in ovarian cancers (7). ERH knockdown blocks the cell cycle procession in the G2/M phase (8); this is especially obvious in human Kirsten rat sarcoma viral oncogene homolog (KRAS) mutation-related tumors (such as colorectal, lung and pancreatic cancer) (9).

The normal-winged alleles of the rudimentary locus of *Drosophila melanogaster* encodes a protein possessing the first three enzymatic activities of the pyrimidine biosynthesis pathway (10). Mutations in the rudimentary gene are manifested in a characteristic truncation of the wings, and the severity of the wing truncation is thought to reflect the level of rudimentary gene expression (11). Drosophila enhancer of rudimentary (DROER) is the enhancer of rudimentary locus of *Drosophila melanogaster*. ERH gene, which was mapped to chromosome 1 band 7q34 by fluorescence *in situ* hybridization in humans, shares a high sequence identity with DROER (12). Corresponding gene and protein analogues of ERH can be found in various species (13), such as flowering plants (14, 15) (*Arabidopsis thaliana*), nematodes (*Caenorhabditis elegans*), and insects (*Aedes aegypti*). Lower vertebrates (*Zebrafish*), mammals (*Mus musculus*), and humans (*Homo sapiens*) also have a high degree of sequence conservation. ERH is not found in the fungi, except for the fission yeasts *Schizosaccharomyces, S. pombe, S. octosporus, S. cryophilus*, and *S. japonicus* (16, 17). It was named ERH in human, DROER in Drosophila, and Xenopus homologue of DROER (XERH) in Xenopus and erh in the other non-human species. In vertebrates, human and mouse erh proteins are exactly the same, and there is only one amino acid (isoleucine-valine) difference from zebrafish; DROER is 76% identical to human and mouse erh proteins, and is 49% similar to nematodes and 40% similar to flowering plants (13).

## The Expression, Structure and Distribution of the ERH

**Figure 1:**
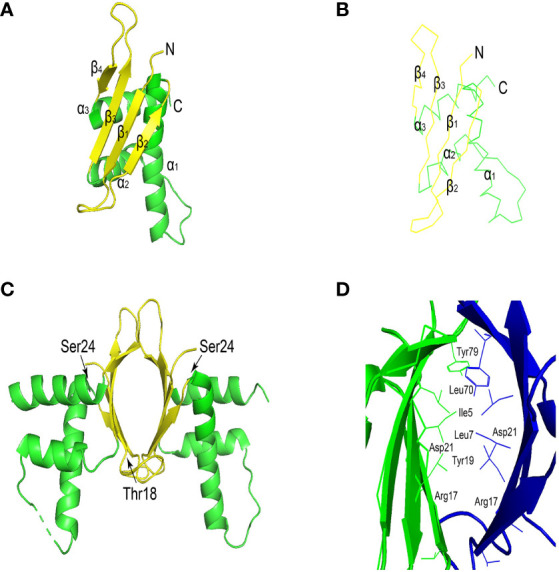
**(A)** Ribbon view of ERH monomer. The α-helices and the β-strands are shown in red and in yellow, respectively. **(B)** View of the main-chain structures in superimposition of the three ERH monomers in the asymmetric unit. Chains **(A–C)**, are colored red, blue, and green, respectively. The superimposition was carried out with the program lsqkab (Kabsch 1976) in CCP4. **(C)** The conserved casein kinase II (CKII) phosphorylation sites (Thr18 and Ser24) are shown as a blue stick model. **(D)** Ribbon view of the interface of the ERH dimer. Cited **Figures 1A–D** (18, 19). This image was generated using PyMOL Molecular Graphics System, Version 2.0 Schrödinger, LLC.

ERH protein sequence is highly conserved. ERH protein has 25 hydrophobic amino acid positions, 27 in DROER, 23 in nematodes, and 20 in Arabidopsis. These hydrophobic amino acids are mostly present in 3 conservative α helices (**Figure 1A**), which is inferred to be an active domain (14). ERH monomeric structure comprises a single domain, and it presents a dimeric structure through 2 beta-sheet regions interacting in the crystal structure (**Figures 1A, B**) (18, 20). There are two conserved casein kinase II (CKII) phosphorylation sites in the ERH protein (**Figure 1C**) (13). It is speculated (21) that CKII can change the secondary structure of Enhancer of rudimentary (ER), thereby adjusting the activity of ER, which is confirmed in structural studies (**Figure 1D**). The overall topology of ERH protein is β1-310-β2-α1-α2-β3-β4-α3 (6).

The mRNA of ERH is expressed maternally, enriched in ectodermal derivatives during development and ubiquitously detectable in adults (22). The XERH is expressed ubiquitously in adult frogs, and the ER transcript is present in the egg and at an increased level during organogenesis; it has been detected in tissues derived from the ectoderm (23). The human ER transcript was found in many normal tissues, including the fetus (12). The XERH protein is distributed in the cytoplasm with only minute amounts in the nucleus (23), but another study (24) showed that the ERH protein can interact with nuclear protein FCP1 and distributed in nucleus, contrary to a previous XERH study. ERH was later shown to localize in both the nucleus and nucleolus in human cells (25).

## ERH Plays a Role in Cancer

According to our review of relevant previous studies, the ERH gene is more expressed in bladder cancer than in normal bladder tissue, and promotes proliferation as well as inhibits cell death (26). Further study showed that ERH can regulate the expression of myelocytomatosis (MYC)gene to have an effect in the migration and invasion of T24 and 5637 bladder cancer cells (27). The study by Balic J *et al.* (28) showed that the combination of ERH and Pontin acts on signal transducer and activator of transcription-3 (STAT3) to enhance the transcriptional activation of its target genes in gastric cancer cells. Pontin is a highly conserved member of the ATPase family, which plays a vital role in the phosphorylation of tryptophan 2 and transcription extension of RNA Pol II (28). Activated STAT3 can interact with other transcription factors (such as STAT1, c-Jun/c-Fos), and can also induce the expression of other transcription factors (such as MYC) to indirectly affect cell transcription (29). STAT3 can combine with Nuclear Factor-κB (NF-κB) to drive unique transcription signals, including genes related to carcinogenesis and immunity (30). These results are consistent with our later studies (31). Our studies showed that ERH gene could affect the apoptosis of bladder cancer T24 cells through the toll-like receptor (TLR), NF-κB, tumor necrosis factor (TNF) or transforming growth factor-β (TGF-β) signaling pathways, and could be a regulator of kinase tyrosine receptor ligand (KITLG) and an activator of malignant tumors growth (32). In 2020, Zhang D *et al.* (33) found that ERH can regulate the epithelial-mesenchymal transition (EMT) to affect cell proliferation, apoptosis, invasion and migration in ovarian cancer cells.

## ERH Acts as a Protein Partner in Cells

### Dimerization Cofactor of Hepatocyte Nuclear Factor-1/Pterin-4α-Carbinolamine Dehydratase

**Figure 2:**
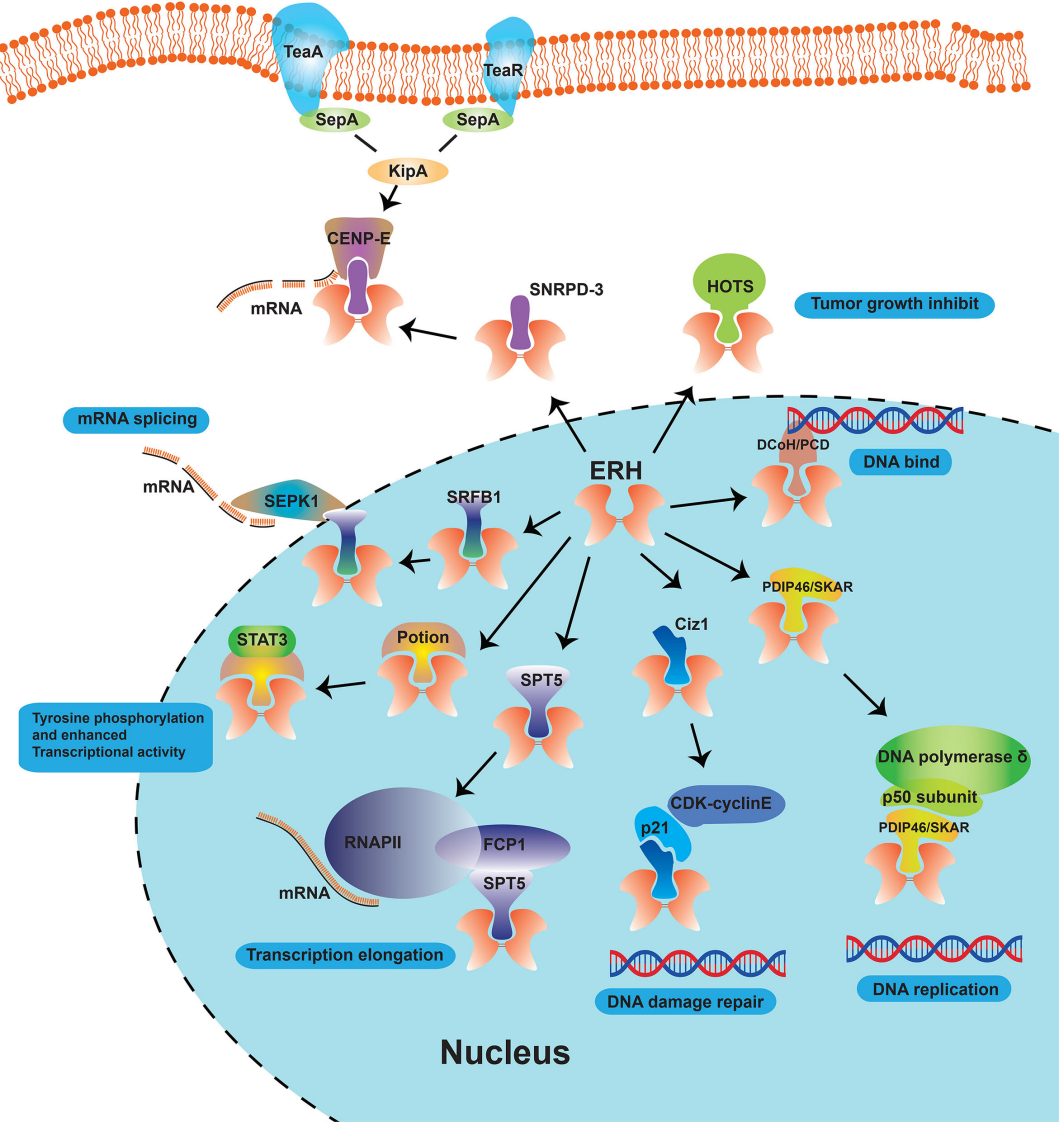
Schematic diagram of the role of ERH proteins as molecular partners in cells. The ERH protein, as a nuclear protein, can interact with many proteins such as DCoH/PCD, SPT5, PDIP46/SKAR, Ciz1, HOTS, SNRPD-3 and DEPDC1B, and involved in many cellular functions like “mRNA solicing”, “Tyrosine phosphorylation and enhanced Transcriptional activity”, “Transcription elongation”, “DNA damage repair”, etc.

Analyzed by Yeast Two-Hybrid (YTH) Assay, Pogge et al. (23) demonstrated that ERH is a protein partner of DCoH/PCD, which is related to the DNA-binding domain of galectin 4 (GAL4) transcription factor (34) and regulates cell growth (**Figure 2**). They found partial co-localization of ERH and DCoH/PCD proteins, and that ERH acts as a transcriptional repressor in a cell type-specific manner upon recruitment to the deoxyribonucleic acid (DNA) *via* its interaction partner DCoH/PCD or by the DNA-binding domain of the GAL4 transcription factor (23). But they did not delve into the specific sites where the two proteins bind, and the report lacked direct evidence of binding. As ERH is a partner of dimerization co-factor of HNF-1 and which is involved in the cell development and regulation in many kinds of cancers, ERH may become a therapeutic target to inhibit the HNF-1 expression.

### Ty Homolog-5 (SPT5)

Analyzed by Co-Immunoprecipitation (Co-IP) and mass spectroscopy (MS), Kwak et al. found that ERH is associated with SPT5 elongation factor (**Figure 2**), which is the phosphorylation target of cyclin dependent kinase 9 (CDK9)/cyclin T1 and are important in regulating the elongation process (35). They further confirmed by co-immunoprecipitation that ERH can specifically bind to F-cell production 1 (FCP1), a phosphatase specific to the carboxyl-terminal domain of the large subunit of RNA polymerase II (RNAPII), stimulating transcription elongation (24, 35). However, the binding site has not been further confirmed. SPT5 is an overexpressing transcription elongation factors and stabilizes RNA polymerase II, orchestrates transcription cycles, and maintains the enhancer landscape (36, 37).

### Polymerase Delta Interaction Protein 46/S6K1 Aly/REF-Like Target

Analyzed by YTH screening, Smyk et al. (38) demonstrated in 2006 that ERH interacted with polymerase δ interacting protein 46 or S6K1 Aly/REF-like target (PDIP46/SKAR, **Figure 2**), a protein partner of both the p50 subunit of DNA polymerase δ and p70 ribosomal protein S6 kinase 1 (S6K1). They examined the interaction between ERH and PDIP46/SKAR by glutathione S-transferase (GST) pull-down, co-IP, MS, and intracellular localizations assays. They further analyzed that 2 regions (274-368 and 379-421, C-terminal) of PDIP46/SKAR interact with ERH. They inferred that ERH connects PDIP46/SKAR with SPT5 and FCP1 and then play roles in coupling transcription to pre-mRNA processing. Blockade of binding to SPT-5 may become an entry point for ERH-targeted therapy.

### Cyclin Dependent Kinase Inhibitor 1A Interacting Zinc Finger Protein 1 (Ciz1)

It is inferred that phosphorylation of CKII sites (Thr18 and Ser24) would disrupt the dimerization of ERH and then disrupt its interaction with other proteins (38). In 2008, using the YTH system, GST, and MS, Lukasik A *et al.* (39) found another molecular chaperone of ERH protein, the zinc finger protein 1 (Ciz1, **Figure 2**), that interacted with p21^Cip1/Waf1^. A region of 114 amino acids comprising residues 531–644 (contains a zine finger motif, 595-617) was shown to interact with ERH using YTH screening. They demonstrated by fluorescence co-localization assay that when Ciz1 and ERH are co-expressed in HeLa cells, Ciz1 could recruit ERH to the region of DNA replication. They indicated that ERH can block the action of Ciz1, and then reduce the expression of ERH inducted by DNA damage, which facilitates CDK-cyclinE-p21^Cip1/Waf1^ complex formation and enables the repair of DNA damage.

### Histocompatibility 19 Opposite Tumor Suppressor

Analyzed by co-IP and MS in 2011, ERH was shown to interact with HOTS, a tumor growth inhibitor encoded by H19 antisense transcript (**Figure 2**) (25). Onyango et al. immunoprecipitated the HOTS- Green fluorescent protein (GFP) by ERH antibodies, demonstrating that native ERH interacts with HOTS protein in HEK293 cells. HOTS is a tumor growth inhibitor, and the overexpression of HOTS inhibits Wilms, rhabdoid, rhabdomyosarcoma, and choriocarcinoma tumor cell growth (25).

### Small Nuclear Ribonucleoprotein D3 Polypeptide

ERH was shown using stable isotope labeling by amino acids in cell culture MS that it can interact with Sm protein SNRPD-3 (**Figure 2**) (9). Co-IP was used to confirm the interaction between ERH and SNRPD-3. ERH is required for the expression of mRNA splicing and the expression of centromere protein E (CENP-E). Xiao et al. (40) found that knockdown of ERH is cell cycle was blocked in the G1 phase in melanoma cells. This indicates that it might be a well target for cell cycle inhibition.

### Dishevelled, EGL-10 and Pleckstrin Domain Containing 1B

In 2015, Wu *et al.* (41) found that ERH can specifically bond with DEP domain containing 1B (DEPDC1B, **Figure 2**) (37). The DEP domain is a protein motif composed of nearly 100 amino acids found in three proteins (Dishevelled, EGL-10 and Pleckstrin), with cell membrane positioning, signal transduction and other functions. ERH was shown to intact with Scaffold attachment factor B1 (SAFB1) to reverse the inhibitory effect on the splicing kinase named Ser/Arg (SR)-rich splicing factor (SRSF) protein kinase 1 (SRPK1) exerted by SAFB1 in mammalian cells (42). DEPDC1B regulates the Rac1/PAK1 signaling and has an effect on the cell proliferation in prostate and pancreatic cancer cells (43, 44).

### Pontin

It has been proven that ERH can combine with Pontin, a highly conserved AAA+ adenosine-triphosphate enzyme (ATPase) family member, to have an effect on serine-phosphorylated STAT3, regulating canonical tyrosine phosphorylation and enhancing transcriptional activity in gastric cancers (28). ERH can interact with thyroid hormone receptor-associated protein 3 (THRAP3), DiGeorge Syndrome Critical Region 8 (DGCR8), protein arginine methyltransferase 1 (PRMT1) and chromatin target of PRMT1 (CHTOP) proteins to regulated mRNA splicing (**Figure 2**) (45).

### Involved in PID-3, ERH-2, TOFU-6, and IFE-3 Small RNA Complex

ERH is involved in many protein complexes, as supported by many research studies. For instance, Perez-Borrajero *et al.* (46) found that ERH was involved in the complex PETISCO (PID-3, ERH-2, TOFU-6, and IFE-3 small RNA complex), which is required for 21U RNA biogenesis. Another study showed that ERH is involved in the complex PICS (piRNA biogenesis and chromosome segregation, contains TOFU-6, PID-1, PICS-1, TOST-1 and ERH-2), which is concentrated at the perinuclear granule zone and engages in piDNA processing and chromosome segregation (**Figure 2**) (47).

### Others

ERH is reported to interact with some other proteins (48), such as mediator complex subunit 31/comparative gene identification protein 125 (MED31/CGI-125), tumor protein p53 (TP53), 70 kDa heat shock protein 8 (heat shock protein, HSPA8) in Li-Fraumeni syndrome, but these results have not been confirmed in experiments.

## Functions of ERH Gene

### Pyrimidine Metabolism

**Figure 3:**
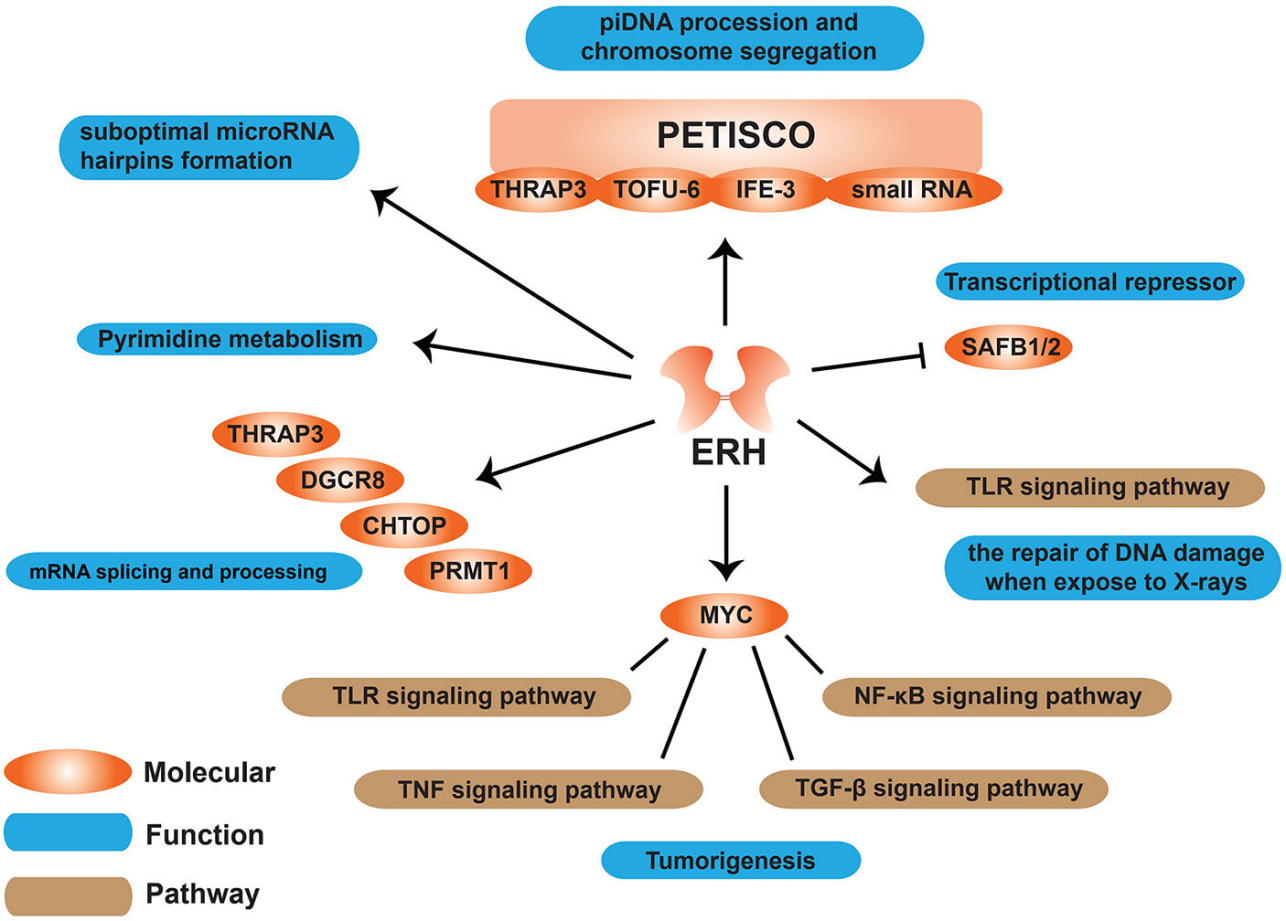
Schematic diagram of functions ERH protein involved in cells. ERH protein can affect many molecules like MYC, SAFB1/2, and some pathways to have an effect on some functions on “tumorigenesis”, “mRNA splicing and processing”, “pyrimidine metabolism” etc.

Literature has shown that ERH plays an important role in biological processes such as pyrimidine biosynthesis, cell cycle regulation, and transcription inhibition (6). Enhanced mutation can promote the expression of carbamoyl phosphate synthetase II (CPSase), aspartate transcarbamoylase (ATCase), and dihydroorotase (DHOase) (CAD), a multifunctional enzyme involved in *de novo* pyrimidine biosynthesis (16). Interestingly, as Smyk *et al.* (38) speculated, if ERH is indeed involved in cell growth control, mutations in ERH can increase the effect of rudimentary mutations, resulting in enhanced wing truncation without directly affecting pyrimidine metabolism (38). No further studies have found whether ERH is directly or indirectly related to pyrimidine metabolism (**Figure 3**).

### Transcriptional Repressor

ERH acts as a transcriptional repressor (34). ERH interacts with SAFB1 and alleviates the inhibition that the SAFB1/2 proteins exert on SRPK1, but it does not affect SAFB1/2 function in transcription (**Figure 3**).

### Cell Cycle Regulation

As the ERH protein can be phosphorylated by CKII, which is a kinase required at the G1/S and G2/M transitions, it is suggested that ERH protein may be involved in cell cycle regulation (38). Analyzed by luciferase reporter assay, Ishikawa et al. (41) demonstrated that miR-574-3p can bind to and regulate ERH to have an effect on the repair of DNA damage. It has been reported that ERH can interact with CIZ1 to initiate the process of DNA replication (39). CIZ1 is a zinc finger protein that can interact with p21, an important CDK2 inhibitor. As centromere-associated protein E (CENP-E), who degraded on mitosis exit and resynthesized in the next S-phase, can be inhibited by the combination of ERH and SNRPD-3. ERH has been shown to regulate the cell cycle in G2/M-phase (8, 9, 49). Cells lacking ERH do not complete DNA replication after release from a replication block (45). ERH is only weakly expressed in undivided hepatocyte cell lines, while it is expressed in large amounts in fibroblasts and hepatocarcinoma cell lines, indicating that ERH may have functions necessary for cell proliferation (7, 13).

### DNA Damage Repair

It was shown that loss of ERH attenuated UV-induced DNA damage repair in hepatocellular carcinoma (HCC) cells (50). Ishikawa *et al.* (41) discovered for the first time that in human lung adenocarcinoma, cerebral medulloblastoma, and astrocytoma cells, ERH is related to the repair of DNA damage when exposed to X-rays. ERH can control the expression of ATR (ataxia telangiectasia-mutated and Rad3-related) to regulate ATR-signaling pathway, which is a major mechanism by which cells respond to and repair replication-associated damage (45) (**Figure 3**).

### mRNA Splicing

It is demonstrated that ERH regulates mRNA splicing of CENP-E mRNA through interaction with the splice protein SNRPD3 (9); other studies have demonstrated that ERH can interact with thyroid hormone receptor-associated protein 3 (THRAP3), DGCR8, CHTOP and PRMT1 proteins, and most of these proteins are involved in mRNA splicing and processing (49) (**Figure 3**).

### MicroRNA Clustering Assists in Processing of Suboptimal MicroRNA Hairpins

It was reported in 2020 by Fang W *et al.* (51) that ERH protein is involved in the processing of suboptimal microRNA hairpin formation. SAFB can both dimerize and interact with ERH, and similarly, ERH can both dimerize and interact with Microprocessor. Together, these two proteins might mediate the association of two or more Microprocessors (51).

### Meiosis

Some studies on the protein erh1 in yeast suggest some potential functions of ERH. Yamashita A *et al.* (52) found that *S. pombe* strains that are deficient in erh1 have significantly reduced tolerance to low temperature and participate in the Mmi1/DSR process, which causes the degradation of meiotic transcripts and is deleterious for meiosis (53, 54). In addition, Erh1 and its molecular chaperone can target and mediate histone 3 lysine 9 (H3K9) methyltransferase Clr4 to assemble facultative heterochromatin during meiosis (55, 56). Erh1 is proven to bind with protein Mmi1 to form a stoichiometric complex, called the Erh1-Mmi1 complex (EMC), to promote meiotic mRNA decay and facultative heterochromatin assembly (57). Mmi1 has a YTH domain, which can bind to target RNA, and the amino terminal end (95-122) of Mmi1 can bind to the processing complex of Erh1 and RNA, and finally form EMC (54), which is critical for nuclear retention of meiotic mRNAs (58).

### Erythroid Differentiation

*ERH* gene was found (59) to be continuously regulated during erythropoiesis and its expression increased during differentiation, indicating that it plays an important role in red blood cell differentiation. But no further studies were conducted on this topic.

## Molecules that Regulate ERH Expression

The expression of ERH is affected by many factors. In 2014, Ishikawa *et al.* (41) found that when some malignant cells are exposed to X-rays, they can induce the expression of miR-574-3p, which suppresses the production of the ERH protein, resulting in the inhibition of cell growth. It was demonstrated by Sutherland J *et al.* (60) that Musashi-1 has the function of upregulating ERH expression. ERH is highly conserved and stable in its structure. It is an ideal therapeutic target for tumor-targeted therapeutic drugs.

## Is *ERH* a Good Target for Drug Design?

Many studies suggest that ERH may be a good target for tumor therapy, and there are a few drugs that have been found to already target ERH for cancer therapy. However, it might cause a lot of side effects to inhibit the expression of ERH since it fulfills so many different roles. In 2015, Weng et al. (50) used AZD7762 (a CHK1 inhibitor) to inhibit the ERH-ATR axis, and they found that AZD7762 induces S-phase arrest and sensitizes HCC cells to doxorubicin, a well-studied chemotherapy for treating HCC4. They also observed strong inhibition in the growth of HCC xenografts in mice treated with a combination of doxorubicin plus AZD7762 (50). In 2021, Park et al. (61) confirmed that ERH augmented anthocyanins isolated from Meoru (AIMs)-induced caspase-dependent apoptosis by activating caspase-3 and -9. They discussed the relationship between augmented expression of ERH and the therapeutic effects of AIM (61). Whether it is because of the side effects, or whether there is no drug that can inhibit ERH, still needs further confirmation. According to the existing research results, by affecting the CKII phosphorylation sites of *ERH* gene, it may affect the binding of ERH protein to its protein partners, thereby affecting the further functions to inhibit the development of malignancies.

## Conclusion

The *ERH* gene encodes a nuclear protein that is highly conserved in animals and plants. Recent studies have shown that ERH plays an adjunct role in promoting tumorigenesis in a variety of malignancies. ERH protein can be combined with a variety of proteins, affecting cell proliferation, cell cycle, DNA repair and other functions of different directions. The *ERH* gene plays an important role in the occurrence and development of malignant tumors, however, there are only a few drugs that target and regulate the expression of ERH by now. For the treatment of the majority of cancer patients, more targeted drugs to inhibit ERH expression should be developed.

## Author Contributions

Conceptualization: KP, M-LL, Z-SC and C-HH. Literature review: LH, Z-DS, BC, Y-YM, HX and DP. Quality Control: Z-SC and C-HH. Language editing: HF. Writing—original draft preparation: KP, M-LL, HF. Writing—review and editing: Z-SC and C-HH. Supervision, Z-SC and C-HH. All authors contributed to the article and approved the submitted version.

## Funding

National Natural Science Fund (82004100, 81774089); Jiangsu Maternal and Child Health Association Project (FYX202026); Jiangsu Province key research and development program (BE2020758, BE2019637); Xuzhou Medical University Excellent Talent Fund Project (XYFY2020016, XYFY2020026); Jiangsu Province, the medical innovation team (CXTDA2017048).

## Conflict of Interest

The authors declare that the research was conducted in the absence of any commercial or financial relationships that could be construed as a potential conflict of interest.

## Publisher’s Note

All claims expressed in this article are solely those of the authors and do not necessarily represent those of their affiliated organizations, or those of the publisher, the editors and the reviewers. Any product that may be evaluated in this article, or claim that may be made by its manufacturer, is not guaranteed or endorsed by the publisher.

## References

[1] Siegel RL Miller KD. Cancer Statistic. CA Cancer J Clin (2022) 72:7–33. doi: 10.3322/caac.21708 35020204

[2] Kang Y Jin Y Li Q Yuan X. Advances in Lung Cancer Driver Genes Associated With Brain Metastasis. Front Oncol (2020) 10:606300. doi: 10.3389/fonc.2020.606300 33537237 PMC7848146

[3] Danesi R Fogli S Indraccolo S Del Re M Dei Tos AP Leoncini L. Druggable Targets Meet Oncogenic Drivers: Opportunities and Limitations of Target-Based Classification of Tumors and the Role of Molecular Tumor Boards. ESMO Open (2021) 6:100040. doi: 10.1016/j.esmoop.2020.100040 33540286 PMC7859305

[4] Chen ZY Hsieh YM Huang CC Tsai CC. Inhibitory Effects of Probiotic Lactobacillus on the Growth of Human Colonic Carcinoma Cell Line HT-29. Molecules (2017) 22(1):107. doi: 10.3390/molecules22010107 PMC6155858 28075415

[5] Chiang CM Chang YJ Wu JY Chang TS. Production and Anti-Melanoma Activity of Methoxyisoflavones From the Biotransformation of Genistein by Two Recombinant Escherichia Coli Strains. Molecules (2017) 22(1):87. doi: 10.3390/molecules22010087 PMC6155705 28054996

[6] Jin T Guo F Serebriiskii IG Howard A Zhang YZ. A 1.55 A Resolution X-Ray Crystal Structure of HEF2/ERH and Insights Into Its Transcriptional and Cell-Cycle Interaction Networks. Proteins (2007) 68:427–37. doi: 10.1002/prot.21343 17444515

[7] Zafrakas M Losen I Knuchel R Dahl E. Enhancer of the Rudimentary Gene Homologue (ERH) Expression Pattern in Sporadic Human Breast Cancer and Normal Breast Tissue. BMC Cancer (2008) 8:145. doi: 10.1186/1471-2407-8-145 18500978 PMC2426700

[8] Fujimura A Kishimoto H Yanagisawa J Kimura K. Enhancer of Rudimentary Homolog (ERH) Plays an Essential Role in the Progression of Mitosis by Promoting Mitotic Chromosome Alignment. Biochem Biophys Res Commun (2012) 423:588–92. doi: 10.1016/j.bbrc.2012.06.018 22704934

[9] Weng MT Lee JH Wei SC Li Q Shahamatdar S Hsu D. Evolutionarily Conserved Protein ERH Controls CENP-E mRNA Splicing and Is Required for the Survival of KRAS Mutant Cancer Cells. Proc Natl Acad Sci USA (2012) 109:E3659–67. doi: 10.1073/pnas.1207673110 PMC3535619 23236152

[10] Falk DR Mccaughin G Cogley T. A Genetic and Biochemical Analysis of the Temperature Sensitive, Normal-Winged Alleles of the Rudimentary Locus of Drosophila Melanogaster. Genetics (1977) 86:765–77. doi: 10.1093/genetics/86.4.765 PMC1213709 21832

[11] Fausto-Sterling A Hsieh L. Studies on the Female-Sterile Mutant Rudimentary of Drosophila Melanogaster. 1. An Analysis of the Rudimentary Wing Phenotype. Dev Biol (1976) 51:269–81. doi: 10.1016/0012-1606(76)90143-3 821800

[12] Isomura M Okui K Fujiwara T Shin S Nakamura Y. Cloning and Mapping of a Novel Human cDNA Homologous to DROER, the Enhancer of the Drosophila Melanogaster Rudimentary Gene. Genomics (1996) 32:125–7. doi: 10.1006/geno.1996.0086 8786099

[13] Gelsthorpe M Pulumati M Mccallum C Dang-Vu K Tsubota SI. The Putative Cell Cycle Gene, Enhancer of Rudimentary, Encodes a Highly Conserved Protein Found in Plants and Animals. Gene (1997) 186:189–95. doi: 10.1016/S0378-1119(96)00701-9 9074495

[14] Schneider K Wells B Dolan L Roberts K. Structural and Genetic Analysis of Epidermal Cell Differentiation in Arabidopsis Primary Roots. Development (1997) 124:1789–98. doi: 10.1242/dev.124.9.1789 9165126

[15] Wang W Yang X Tangchaiburana S Ndeh R Markham JE Tsegaye Y. An Inositolphosphorylceramide Synthase Is Involved in Regulation of Plant Programmed Cell Death Associated With Defense in Arabidopsis. Plant Cell (2008) 20:3163–79. doi: 10.1105/tpc.108.060053 PMC2613663 19001565

[16] Wojcik E Murphy AM Fares H Dang-Vu K Tsubota SI. Enhancer of Rudimentaryp1, E(R)P1, A Highly Conserved Enhancer of the Rudimentary Gene. Genetics (1994) 138:1163–70. doi: 10.1093/genetics/138.4.1163 PMC1206255 7896098

[17] Krzyzanowski MK Kozlowska E Kozlowski P. Identification and Functional Analysis of the Erh1(+) Gene Encoding Enhancer of Rudimentary Homolog From the Fission Yeast Schizosaccharomyces Pombe. PloS One (2012) 7:e49059. doi: 10.1371/journal.pone.0049059 23145069 PMC3492181

[18] Arai R Kukimoto-Niino M Uda-Tochio H Morita S Uchikubo-Kamo T Akasaka R. Crystal Structure of an Enhancer of Rudimentary Homolog (ERH) at 2.1 Angstroms Resolution. Protein Sci (2005) 14:1888–93. doi: 10.1110/ps.051484505 PMC2253356 15937287

[19] Berman HM Westbrook J Feng Z Gilliland G Bhat TN Weissig H. The Protein Data Bank. Nucleic Acids Research (2000) 28:235–42. 10.1093/nar/28.1.235 PMC102472 10592235

[20] Sehnal D Bittrich S Deshpande M Svobodová R Berka K Bazgier V. Mol* Viewer: Modern Web App for 3D Visualization and Analysis of Large Biomolecular structures. Nucleic Acids Res (2021) 49(W1):W431–37. doi: 10.1136/bmjopen-2019-036563 PMC8262734 33956157

[21] Wan C Tempel W Liu ZJ Wang BC Rose RB. Structure of the Conserved Transcriptional Repressor Enhancer of Rudimentary Homolog. Biochemistry (2005) 44:5017–23. doi: 10.1021/bi047785w 15794639

[22] Jin T Howard A Golemis EA Wang Y Zhang YZ. Overproduction, Purification, Crystallization and Preliminary X-Ray Diffraction Studies of the Human Transcription Repressor ERH. Acta Crystallogr Sect F Struct Biol Cryst Commun (2005) 61:531–3. doi: 10.1107/S1744309105012388 PMC1952306 16511088

[23] Pogge Von Strandmann E Senkel S Ryffel GU. ERH (Enhancer of Rudimentary Homologue), a Conserved Factor Identical Between Frog and Human, is a Transcriptional Repressor. Biol Chem (2001) 382:1379–85. doi: 10.1515/BC.2001.170 11688721

[24] Amente S Napolitano G Licciardo P Monti M Pucci P Lania L. Identification of Proteins Interacting With the RNAPII FCP1 Phosphatase: FCP1 Forms a Complex With Arginine Methyltransferase PRMT5 and It Is a Substrate for PRMT5-Mediated Methylation. FEBS Lett (2005) 579:683–9. doi: 10.1016/j.febslet.2004.12.045 15670829

[25] Onyango P Feinberg AP. A Nucleolar Protein, H19 Opposite Tumor Suppressor (HOTS), Is a Tumor Growth Inhibitor Encoded by a Human Imprinted H19 Antisense Transcript. Proc Natl Acad Sci USA (2011) 108:16759–64. doi: 10.1073/pnas.1110904108 PMC3189046 21940503

[26] Pang K Lv Q Ning SY Hao L Shi ZD Zang GH. ERH Is Up-Regulated in Bladder Cancer and Regulates the Proliferation and Apoptosis of T24 Bladder Cancer Cells. Int J Of Clin And Exp Med (2017) 10:15269–77.

[27] Pang K Zhang Z Hao L Shi Z Chen B Zang G. The ERH Gene Regulates Migration and Invasion in 5637 and T24 Bladder Cancer Cells. BMC Cancer (2019) 19:225. doi: 10.1186/s12885-019-5423-9 30866868 PMC6417071

[28] Balic JJ Garama DJ Saad MI Yu L West AC West AJ. Serine-Phosphorylated STAT3 Promotes Tumorigenesis *via* Modulation of RNA Polymerase Transcriptional Activity. Cancer Res (2019) 79:5272–87. doi: 10.1158/0008-5472.CAN-19-0974 31481496

[29] Jones SA Jenkins BJ. Recent Insights Into Targeting the IL-6 Cytokine Family in Inflammatory Diseases and Cancer. Nat Rev Immunol (2018) 18:773–89. doi: 10.1038/s41577-018-0066-7 30254251

[30] Yang J Liao X Agarwal MK Barnes L Auron PE Stark GR. Unphosphorylated STAT3 Accumulates in Response to IL-6 and Activates Transcription by Binding to NFkappaB. Genes Dev (2007) 21:1396–408. doi: 10.1101/gad.1553707 PMC1877751 17510282

[31] Pang K Han C Hao L Shi Z. ERH Gene Targets NFKB1 Gene and Regulating NF-Kappa B Pathway to Enhances Epithelial-Mesenchymal Transition Related Migration and Invasion of Bladder Cancer T24 Cells. Int J Of Urol (2019), 5–5. WILEY 111 RIVER ST, HOBOKEN 07030-5774, NJ USA.

[32] Pang K Hao L Shi Z Chen B Pang H Dong Y. Comprehensive Gene Expression Analysis After ERH Gene Knockdown in Human Bladder Cancer T24 Cell Lines. Gene (2020) 738:144475. doi: 10.1016/j.gene.2020.144475 32081697

[33] Zhang D Chu YJ Song KJ Chen YL Liu W Lv T. Knockdown of Enhancer of Rudimentary Homolog Inhibits Proliferation and Metastasis in Ovarian Cancer by Regulating Epithelial-Mesenchymal Transition. BioMed Pharmacother (2020) 125:109974. doi: 10.1016/j.biopha.2020.109974 32036222

[34] Pogge V Strandmann E Senkel S Ryffel GU. Ectopic Pigmentation in Xenopus in Response to DCoH/PCD, the Cofactor of HNF1 Transcription Factor/Pterin-4alpha-Carbinolamine Dehydratase. Mech Dev (2000) 91:53–60. doi: 10.1016/S0925-4773(99)00269-5 10704830

[35] Kwak YT Guo J Prajapati S Park KJ Surabhi RM Miller B. Methylation of SPT5 Regulates its Interaction With RNA Polymerase II and Transcriptional Elongation Properties. Mol Cell (2003) 11:1055–66. doi: 10.1016/S1097-2765(03)00101-1 12718890

[36] Han J Tam K Tam C Hollis RP Kohn DB. Improved Lentiviral Vector Titers From a Multi-Gene Knockout Packaging Line. Mol Ther Oncolytics (2021) 23:582–92. doi: 10.1016/j.omto.2021.11.012 PMC8660686 34938858

[37] Wu D Zhu X Jimenez-Cowell K Mold AJ Sollecito CC Lombana N. Identification of the GTPase-Activating Protein DEP Domain Containing 1B (DEPDC1B) as a Transcriptional Target of Pitx2. Exp Cell Res (2015) 333:80–92. doi: 10.1016/j.yexcr.2015.02.008 25704760 PMC4387072

[38] Smyk A Szuminska M Uniewicz KA Graves LM Kozlowski P. Human Enhancer of Rudimentary Is a Molecular Partner of PDIP46/SKAR, A Protein Interacting With DNA Polymerase Delta and S6K1 and Regulating Cell Growth. FEBS J (2006) 273:4728–41. doi: 10.1111/j.1742-4658.2006.05477.x 16984396

[39] Lukasik A Uniewicz KA Kulis M Kozlowski P. Ciz1, a P21 Cip1/Waf1-Interacting Zinc Finger Protein and DNA Replication Factor, Is a Novel Molecular Partner for Human Enhancer of Rudimentary Homolog. FEBS J (2008) 275:332–40. doi: 10.1111/j.1742-4658.2007.06203.x 18081865

[40] Martin LB Liebl AL Kilvitis HJ. Covariation in Stress and Immune Gene Expression in a Range Expanding Bird. Gen Comp Endocrinol (2015) 211:14–9. doi: 10.1016/j.ygcen.2014.11.001 25448257

[41] Ishikawa K Ishikawa A Shoji Y Imai T. A Genotoxic Stress-Responsive miRNA, miR-574-3p, Delays Cell Growth by Suppressing the Enhancer of Rudimentary Homolog Gene *In Vitro* . Int J Mol Sci (2014) 15:2971–90. doi: 10.3390/ijms15022971 PMC3958894 24566139

[42] Drakouli S Lyberopoulou A Papathanassiou M Mylonis I Georgatsou E. Enhancer of Rudimentary Homologue Interacts With Scaffold Attachment Factor B at the Nuclear Matrix to Regulate SR Protein Phosphorylation. FEBS J (2017) 284:2482–500. doi: 10.1111/febs.14141 28627136

[43] Li Z Wang Q Peng S Yao K Chen J Tao Y. The Metastatic Promoter DEPDC1B Induces Epithelial-Mesenchymal Transition and Promotes Prostate Cancer Cell Proliferation *via* Rac1-PAK1 Signaling. Clin Transl Med (2020) 10:e191. doi: 10.1002/ctm2.191 33135357 PMC7536616

[44] Zhang S Shi W Hu W Ma D. DEP Domain-Containing Protein 1b (DEPDC1B) Promotes Migration and Invasion in Pancreatic Cancer Through the Rac1/PAK1-LIMK1-Cofilin1 Signaling Pathway. Onco Targets Ther (2020) 13:1481–96. doi: 10.2147/OTT.S229055 PMC7035893 32110046

[45] Kavanaugh G Zhao R Guo Y Mohni KN Glick G Lacy ME. Enhancer of Rudimentary Homolog Affects the Replication Stress Response Through Regulation of RNA Processing. Mol Cell Biol (2015) 35:2979–90. doi: 10.1128/MCB.01276-14 PMC4525316 26100022

[46] Oehl-Jaschkowitz B Vanakker OM De Paepe A Menten B Martin T Weber G. Deletions in 14q24.1q24.3 Are Associated With Congenital Heart Defects, Brachydactyly, and Mild Intellectual Disability. Am J Med Genet A (2014) 164a:620–6. doi: 10.1002/ajmg.a.36321 24357125

[47] Zeng C Weng C Wang X Yan YH Li WJ Xu D. Functional Proteomics Identifies a PICS Complex Required for piRNA Maturation and Chromosome Segregation. Cell Rep (2019) 27:3561–3572.e3. doi: 10.1016/j.celrep.2019.05.076 31216475

[48] Gelsthorpe ME Tan Z Phillips A Eissenberg JC Miller A Wallace J. Regulation of the Drosophila Melanogaster Protein, Enhancer of Rudimentary, by Casein Kinase II. Genetics (2006) 174:265–70. doi: 10.1534/genetics.106.061465 PMC1569772 16849599

[49] Weng MT Luo J. The Enigmatic ERH Protein: Its Role in Cell Cycle, RNA Splicing and Cancer. Protein Cell (2013) 4:807–12. doi: 10.1007/s13238-013-3056-3 PMC4875447 24078386

[50] Weng MT Tung TH Lee JH Wei SC Lin HL Huang YJ. Enhancer of Rudimentary Homolog Regulates DNA Damage Response in Hepatocellular Carcinoma. Sci Rep (2015) 5:9357. doi: 10.1038/srep09357 25880358 PMC4399501

[51] Fang W Bartel DP. MicroRNA Clustering Assists Processing of Suboptimal MicroRNA Hairpins Through the Action of the ERH Protein. Mol Cell (2020) 78:289–302.e6. doi: 10.1016/j.molcel.2020.01.026 32302541 PMC7243034

[52] Yamashita A Takayama T Iwata R Yamamoto M. A Novel Factor Iss10 Regulates Mmi1-Mediated Selective Elimination of Meiotic Transcripts. Nucleic Acids Res (2013) 41:9680–7. doi: 10.1093/nar/gkt763 PMC3834831 23980030

[53] Harigaya Y Tanaka H Yamanaka S Tanaka K Watanabe Y Tsutsumi C. Selective Elimination of Messenger RNA Prevents an Incidence of Untimely Meiosis. Nature (2006) 442:45–50. doi: 10.1038/nature04881 16823445

[54] Hazra D Andric V Palancade B Rougemaille M Graille M. Formation of S. Pombe Erh1 Homodimer Mediates Gametogenic Gene Silencing and Meiosis Progression. Sci Rep (2020) 10:1034. doi: 10.1038/s41598-020-57872-4 31974447 PMC6978305

[55] Tashiro S Asano T Kanoh J Ishikawa F. Transcription-Induced Chromatin Association of RNA Surveillance Factors Mediates Facultative Heterochromatin Formation in Fission Yeast. Genes Cells (2013) 18:327–39. doi: 10.1111/gtc.12038 23388053

[56] Zofall M Yamanaka S Reyes-Turcu FE Zhang K Rubin C Grewal SI. RNA Elimination Machinery Targeting Meiotic mRNAs Promotes Facultative Heterochromatin Formation. Science (2012) 335:96–100. doi: 10.1126/science.1211651 22144463 PMC6338074

[57] Sugiyama T Thillainadesan G Chalamcharla VR Meng Z Balachandran V Dhakshnamoorthy J. Enhancer of Rudimentary Cooperates With Conserved RNA-Processing Factors to Promote Meiotic mRNA Decay and Facultative Heterochromatin Assembly. Mol Cell (2016) 61:747–59. doi: 10.1016/j.molcel.2016.01.029 PMC4791967 26942678

[58] Xie G Vo TV Thillainadesan G Holla S Zhang B Jiang Y. A Conserved Dimer Interface Connects ERH and YTH Family Proteins to Promote Gene Silencing. BMC Cancer (2019) 10:251. doi: 10.1038/s41467-018-08273-9 PMC6335422 30651569

[59] Da Cunha AF Brugnerotto AF Duarte AS Lanaro C Costa GG Saad ST. Global Gene Expression Reveals a Set of New Genes Involved in the Modification of Cells During Erythroid Differentiation. Cell Prolif (2010) 43:297–309. doi: 10.1111/j.1365-2184.2010.00679.x 20546246 PMC6496675

[60] Sutherland JM Sobinoff AP Fraser BA Redgrove KA Davidson TL Siddall NA. RNA Binding Protein Musashi-1 Directly Targets Msi2 and Erh During Early Testis Germ Cell Development and Interacts With IPO5 Upon Translocation to the Nucleus. FASEB J (2015) 29:2759–68. doi: 10.1096/fj.14-265868 25782991

[61] Hammond ER Tummala R Berglind A Syed F Wang X Desta B. Study Protocol for the International Systemic Lupus Erythematosus Prospective Observational Cohort Study (SPOCS): Understanding Lupus and the Role of Type I Interferon Gene Signature. BMJ Open (2020) 10:e036563. doi: 10.1136/bmjopen-2019-036563 PMC7467530 32873668

